# Seizing an opportunity

**DOI:** 10.7554/eLife.48336

**Published:** 2019-06-18

**Authors:** Perry Zurn, Danielle S Bassett

**Affiliations:** 1 Department of PhilosophyAmerican UniversityWashingtonUnited States; 2 Department of Bioengineering, School of Engineering & Applied ScienceUniversity of PennsylvaniaPhiladelphiaUnited States; 3 Department of Physics & Astronomy, College of Arts & SciencesUniversity of PennsylvaniaPhiladelphiaUnited States; 4 Department of Electrical & Systems Engineering, School of Engineering & Applied ScienceUniversity of PennsylvaniaPhiladelphiaUnited States; 5 Department of Neurology, Perelman School of MedicineUniversity of PennsylvaniaPhiladelphiaUnited States; 6 Department of Psychiatry, Perelman School of MedicineUniversity of PennsylvaniaPhiladelphiaUnited States

**Keywords:** philosophy of biology, philosophy, literature, network science, history, curiosity

## Abstract

As the techniques available to neuroscientists to probe the inner workings of the brain become more powerful, the volume of data generated increases exponentially and the tools used to analyze these data become ever more sophisticated. Alongside this feverish press into the future comes a growing interest in the use of new data technologies to study old literary and philosophical texts. And as researchers develop new methods to unearth patterns hidden within complex data, it is natural to think of these old texts as simply more data reflecting the intricacies of the human mind, waiting to succumb to the powerful, objective, and reliable perception of machines. Here we argue that historical texts are more than prone booty to be exploited: rather, they offer researchers in neuroscience, philosophy, and literature the opportunity to work together, to learn from and enrich each other’s methodologies. Using the study of human curiosity as an illustration, we describe our vision for a collaborative approach to exploring the mysteries of the mind and human behavior.

When *eLife* asked us to describe the “opportunities that literary and philosophical texts present for neuroscience,” it was difficult to get past the first word: opportunities. According to the *Oxford English Dictionary*, “opportunity” stems from the Latin *ob-portus*. It signifies favorable winds blowing one reliably toward port. Conversely, “inopportune” refers to the unfavorable winds that might whisk one, all pell-mell and helter-skelter, away from harbor. Here we take literature and philosophy not only to present opportunities for neuroscience but to themselves be opportune for neuroscience. They are the winds that can help steer the ship to port; that is, towards answers to classical questions about the nature and function of the mind. But we are, perhaps equally, interested in the inopportunities presented by philosophy and literature. How might they steer neuroscience, and its kindred disciplines of psychology and cognitive science, elsewhere? To a field as yet undefined? To future possibilities yet unknown? To questions not yet asked? In what follows, then, we explore the opportunities and inopportunities that literary and philosophical texts present for the interdisciplinary study of the mind.

Crossing the sea of discovery is an apt metaphor for both the sciences and the humanities. The vastness of the universe and the immeasurable mystery of the human condition seem so far from reach given the difficulties of navigation and the incremental character of our progress. Add to that our uneasy reliance on chance, and the whole project takes on an air of reckless ambition. It’s no wonder that, over the centuries, leading lights have cast both science and philosophy as questionable – if not ludicrous – enterprises. Augustine called philosophy a superfluous activity (*The Confessions,*written circa 397), while Erasmus said that “to measure the stars” is sheer madness (*In Praise of Folly,* 1511). Even Rousseau would claim, in 1751, that all of the arts and sciences are fueled by a certain “vain curiosity” (Rousseau, 2008). It is true that each field, in its own way, attempts to plumb the unplumbable. “The reality we can put into words,” as Werner Heisenberg put it in 1942, “is never reality itself” (Heisenberg, 1998). And yet, insofar as neuroscience, philosophy, and literature are all drawn, irrepressibly, to the mysteries of the mind and human sociality, how might they team up in this plucky project of knowing the unknown? In this gamble of transliterating the mind into formulas, words, and images? Of translating *what is* into *what speaks*?

## Not just another data set

In an age that is increasingly smitten with the beauty of rich patterns hidden within complex data, it is natural to think of literary and philosophical texts as more data simply waiting to succumb to the powerful, objective, and reliable perception of machines. There must be so many clear answers obtainable with seemingly so little effort! The application of computational methods to the traditional disciplines of literature, history, and philosophy is increasing (Underwood, 2019) as digital technologies, algorithms, and software allow researchers in these fields to curate, archive, systematize, search, analyze, mine, and visualize texts in ways that were not possible with their traditional investigative methods. While applied broadly to study culture, politics, and more, the utility of computational textual analysis in neuroscience is increasingly evident. A text is an output from the human mind, and thus it stands to reason that we should be able to read from a text the principles of thought, and that those principles of thought presumably have simple neural underpinnings. Our ability to extract such principles from text is expected due to the nature of science. Scientific inquiry consistently infers principles from observations; just as we can read from leaf vasculature the principles of nutrient flow for which we might seek genetic causes, we should be able to read from text the principles of thought for which we seek neural causes. As the variety of debates surrounding the “digital humanities” (see, for example, Da, 2019) make clear, however, this work is complex and difficult to do well.

And yet, insofar as neuroscience, philosophy, and literature are all drawn, irrepressibly, to the mysteries of the mind and human sociality, how might they team up in this plucky project of knowing the unknown?

Reading the principles of thought: it sounds so simple and yet seems so impossible. Computational analysis is a particular action often performed on data, sometimes to answer a question, and more rarely to purposefully inform a theory. But how do we choose the question? Which data should we use? How do we guide the search? Could answers to these questions be fruitfully informed by the work of other fields? From antiquity, philosophers and literary artists have formalized the unknown they wish to know, chosen what relevant observations to make, and constructed theory from observations. What is more, the texts studied in the humanities are not inanimate materials composed of word particles ready to be submitted to mindless actions, but rather adaptive systems displaying complex behaviors and collective dynamics that offer candidate answers to those beyond-action questions. Rather than seeing philosophy and other humanities as new data fields for neuroscience, neuroscientists should challenge themselves to see them as companion explorers. Such a paradigm shift not only contributes to an interdisciplinary understanding of the mind, but also increasingly highlights the limited explanatory power of each disciplinary purview. Together, more is possible.

## Ecologies of knowledge

Around 350 BC, Aristotle wrote in the *Metaphysics* (Book XII, chapter 7) that the more we come to understand the world, the more we and the world draw closer, until the mind itself is the object of thought. Neuroscience aims to do exactly this: take the mind as an object of thought. And yet, as bearers of (perhaps overly curious) minds ourselves, we know the mind is never alone, and thoughts are not generated in a vacuum. Knowledge is not built by a single mind, apart from others. Nor is it built in a single era, divorced from others. Nor is it built in a single field, tradition, school, or lab, isolated from others. In fact, today it is increasingly critical to recognize that knowledge is not built by humans alone, but also by non-human animals and machines (Humphreys, 2009; Haraway, 2008). Knowledge is, therefore, always already developing within ecologies of knowledge, themselves replete with interlocking, adaptive ecosystems of life, culture, and research (Star, 1995). How might we operationalize this?

Exploring – rather than ignoring – the opportunities presented by philosophy and literature to reframe and refine neuroscientific insights requires two commitments: i) to ecological complexity; ii) to ecological temporality. First, a commitment to ecological complexity requires us to resist the pressure to reproduce academic siloes and monocultures and never stray too far afield (de Sousa Santos, 2016). Emulating the great scientist-philosophers and indigenous seers of old, neuroscientists of a new ilk might eagerly trace vestiges of the mind wherever they can be found, in poems and theoretical treatises as much as in fMRI machines and electrodes. Such a practice is synergistic with calls for more “ecologically valid experiments”, which would complement traditional, hyper-controlled conditions with more natural habitats (Burgess et al., 2006). While it is true that ecologically valid experiments produce more uncertain findings, they ask more robust questions. In this instance, we wonder, why not think of literary and philosophical texts as natural habitats of the mind, so much more complicated, yes, but also more promising?

Emulating the great scientist-philosophers and indigenous seers of old, neuroscientists of a new ilk might eagerly trace vestiges of the mind wherever they can be found, in poems and theoretical treatises as much as in fMRI machines and electrodes.

Second, a commitment to ecological temporality requires us to resist the pressure of the present and the narrative of linear progress. Over the past two millennia, the human mind has not evolved appreciably; it has, however, created literary and philosophical texts that preserve the workings of brilliant minds – as well as brilliant theories of mind. There is therefore no scientific reason why we should not be studying, say, Confucius. In fact, to study the mind through only a decade of data points is a bit like studying tree canopies without trunk or root structures. We find it untenable to assume that once a text is old or a scientist has passed away, they enter the ranks of history and are no longer part of the science of the mind. Much like the scientific consideration of living organisms and fossils, the earth and stars light years away, we posit that a science of the mind ought to account for minds today *and* those of yesteryear. This requires that quite young disciplines such as psychology, neuroscience, and cognitive science work together with the far older disciplines of philosophy, literature, and the arts.

## Modeling curiosity in neuroscience and philosophy

Enamored with the promise of the other’s discipline and its potential to speak to our own, we (a philosopher [PZ] and a physicist and neuroscientist [DSB]) embarked on a collaborative project to model curiosity. Approaching neuroscience and network theory, philosophy and literature, as companion explorers, we asked: “What are the models of curiosity present in classical texts and how might those models be tested through the tools of network science to inform future neuroscience experiments?” We began by canvassing the history of Western thought, tracking the use of curiosity-related terms across English, Greek, Latin, French, and German texts. We identified three models – or kinesthetic signatures – of curiosity: i) the busybody, who collects discrete bits of information; ii) the hunter, who focuses on a specific trajectory of inquiry; and iii) the dancer, who takes leaps of creative imagination (Zurn, 2019).

We then operationalized these kinesthetic signatures by constructing mathematical models of the networks created by a busybody (or a hunter or a dancer) as they build up their knowledge through curious actions. The busybody constructs a loose knowledge network, happy to seek disconnected bits of information like trivia. The hunter constructs a tight knowledge network by seeking information that connects one concept to another in a formal manner. The dancer constructs a disparate and dynamic knowledge network by transiently switching between two types of actions: movements to acquire a bit of information unexpectedly far from their current knowledge, and movements to connect already acquired concepts to one another in unexpected ways. Finally, we tested – and confirmed – the presence of these distinct network configurations in the manner in which humans browse Wikipedia, where network nodes represent links clicked or topics searched for, and where network edges represent the fact that two nodes were browsed in succession (Lydon-Staley et al., 2018; Lydon-Staley et al., 2019). In turn, these preferences for building specific knowledge network architectures inform our ongoing neuroimaging experiments to evaluate the ease or difficulty of learning a given network architecture and the underlying patterns of neural activity that support that learning (Kahn et al., 2018; Tompson et al., 2019).

Over the past two millennia, the human mind has not evolved significantly; it has, however, created literary and philosophical texts that preserve the workings of brilliant minds—as well as brilliant theories of mind.

In addition to taking inspiration from literary and philosophical texts in our investigations into curiosity, ideas from network science, cognitive science, and neuroscience prompted us to rethink what it means to be curious. In contrast to the age-old definition of curiosity as a desire to know – or, more recently, as a drive-state for information – we define curiosity as a practice of building connections, as the principle of knowledge network growth (Bassett, 2020; Zurn and Bassett, 2018). This redefinition provides an important contribution to the nascent field of network epistemology, which has yet to account for either the network structures of curiosity or its role in knowledge network building. And besides pushing us forward, this inquiry also pushed us back, back into the dusty annals of history to track and to trace evidence buried in unexpected places. We rediscovered, for example, John Dewey’s characterization of curiosity as the capacity to make the connections between things explicit (Dewey, 1916), and Adam Smith’s claim that curiosity is the labor of connecting one thing to another (*History of Astronomy,* published posthumously in 1795). The interdigitation of times and disciplines in this project suggests the promise of not only increasing familiarity and appreciation across fields, but collaboration.

## Companion explorers

In a letter to his son, Michael, dated March 8, 1941, JRR Tolkien insists that he give up on the overly romanticized notion of a life partner, a woman who would serve as his “guiding star” (Tolkien, 1941). Instead, Tolkien advises, see a life partner for what they are: “companions in shipwreck.” Here we ask the reader to imagine a ship called Inquiry, afloat on the sea of existence. It is barreling toward the port of Mysteries, whether of the mind, the world, or the indefinable juncture between them. Aboard ship are three curious characters: neuroscience, philosophy, and literature. Companion explorers at one moment, companions in shipwreck the next. They may very well reach port in tandem, but just as often, perhaps, they will sail off unexpectedly in another direction, across as yet unrecognized frontiers of human thought. They might lose their ship altogether and build another in its place. Wherever neuroscience is going, it needn’t go alone.

## Note

This Feature Article is part of the Philosophy of Biology collection.
